# An interchangeable role for kainate and metabotropic glutamate receptors in the induction of rat hippocampal mossy fiber long-term potentiation in vivo

**DOI:** 10.1002/hipo.22460

**Published:** 2015-04-25

**Authors:** James L Wallis, Mark W Irvine, David E Jane, David Lodge, Graham L Collingridge, Zuner A Bortolotto

**Affiliations:** Centre for Synaptic Plasticity, School of Physiology and Pharmacology, University of BristolBristol, United Kingdom

**Keywords:** dentate, CA3, LTP, granule cells

## Abstract

The roles of both kainate receptors (KARs) and metabotropic glutamate receptors (mGluRs) in mossy fiber long-term potentiation (MF-LTP) have been extensively studied in hippocampal brain slices, but the findings are controversial. In this study, we have addressed the roles of both mGluRs and KARs in MF-LTP in anesthetized rats. We found that MF-LTP could be induced in the presence of either GluK1-selective KAR antagonists or group I mGluR antagonists. However, LTP was inhibited when the group I mGluRs and the GluK1-KARs were simultaneously inhibited. Either mGlu1 or mGlu5 receptor activation is sufficient to induce this form of LTP as selective inhibition of either subtype alone, together with the inhibition of KARs, did not inhibit MF-LTP. These data suggest that mGlu1 receptors, mGlu5 receptors, and GluK1-KARs are all engaged during high-frequency stimulation, and that the activation of any one of these receptors alone is sufficient for the induction of MF-LTP in vivo. © 2015 The Authors Hippocampus Published by Wiley Periodicals, Inc.

## INTRODUCTION

Long-term potentiation (LTP) is widely considered to provide a good model system for understanding the synaptic processes involved in information storage in the central nervous system. Most forms of LTP are induced by the synaptic activation of *N*-methyl-d-aspartate receptors (NMDARs) and involve both pre- and postsynaptic modes of expression (Bliss and Collingridge, [Bibr b6],[Bibr b7]). There are, however, also NMDAR-independent forms of LTP as first identified (Harris and Cotman, 1986), and most extensively characterized, at the mossy fiber synapse in the hippocampus (Nicoll and Schmitz, 2005; Jane et al., 2009).

The role of glutamate receptors in the induction of mossy fiber LTP (MF-LTP) is controversial. Initial studies suggested that MF-LTP is induced independently of activation of any ionotropic glutamate receptors (Nicoll and Malenka, 1995). However, subsequent studies identified roles for kainate receptors (KARs) (Bortolotto et al., 1999; Contractor et al., 2001) in MF-LTP induction. Prior to this, metabotropic glutamate receptors (mGluRs) were also shown to be involved in the induction of MF-LTP (Ito and Sugiyama, 1991; Bashir et al., 1993). These findings have not, however, met with universal acceptance. With respect to mGluRs, there are reports that antagonists for these receptors, including the prototypic mGluR antagonist, α-methyl-4-carboxyphenylglycine (MCPG) (Bashir et al., 1993), either block the induction of MF-LTP (Ito and Sugiyama, 1991; Bashir et al., 1993; Yeckel et al., 1999; Itoh et al., 2001; Thompson et al., 2005; Nistico et al., 2011) or have no effect (Manzoni et al., 1994; Hsia et al., 1995; Mellor and Nicoll, 2001; Barnes et al., 2010). Regarding KARs, there is general agreement that these can act as triggers for LTP (Bortolotto et al., 1999; Contractor et al., 2001; Schmitz et al., 2003; Pinheiro et al., 2007; Catches et al., 2012). However, there is disagreement regarding whether the KAR involved contains the GluK1 subunit (Bortolotto et al., 1999; Lauri et al., 2001; More et al., 2004; Nistico et al., 2011) or not (Contractor et al., 2001; Breustedt and Schmitz, 2004).

Most previous studies concerning the role of mGluRs and all previous studies concerning the role of KARs in MF-LTP have been performed in brain slices. Differences in the experimental conditions employed may partly explain the differing results obtained. Indeed, there are several incidences where differences in the experimental conditions can dramatically affect the outcome of the experiments. For example, it was found that increasing the extracellular Ca^2+^ concentration from 2 to 4 mM negated the need for the synaptic activation of GluK1 KARs for the induction of MF-LTP (Lauri et al., 2003). Furthermore, increasing tetanization strength or artificially increasing depolarization levels can result in the induction of LTP through mechanisms independent of KAR activation (Schmitz et al., 2003; Pinheiro et al., 2007). Recently, it was found that the role of GluK1-containing KARs is dependent on the slice orientation, with antagonists showing efficacy in parasagittal but not transverse preparations (Sherwood et al., 2012). These dramatic differences led us to investigate the role of mGluRs and KARs at the hippocampal MF synapse in vivo.

In this study, we observed a slowly developing MF-LTP, in both pentobarbitone- and urethane-anesthetized rats, that was independent of the synaptic activation of NMDARs. This form of LTP, which closely resembled that described previously in vivo (Derrick and Martinez, 1994), was insensitive to a range of mGluR and KAR antagonists delivered by intrahippocampal (i.h.) injection. Furthermore, a combination of either an mGlu1 ((*S*)-(+)-α-amino-4-carboxy-2-methylbenzeneacetic acid; LY367385), or an mGlu5, (2-methyl-6-(phenylethynyl)pyridine; MPEP) receptor antagonist, with a GluK1-containing KAR antagonist ((*S*)-1-(2-amino-2-carboxyethyl)-3-(2-carboxy-5-phenylthiophene-3-yl-methyl)-5-methylpyrimidine-2,4-dione; ACET or (2R*,3S*)-1-(9-iodophenanthrene-3-carbonyl)piperazine-2,3-dicarboxylic acid; UBP161) was also ineffective. However, blockade of both group I mGluR subtypes, using either MCPG or a combination of LY367385 and MPEP, together with blockade of GluK1-containing KARs, using either ACET or UBP161, significantly reduced or completely prevented the induction of MF-LTP. These findings suggest that the activation of either mGlu1 receptors or mGlu5 receptors or GluK1-containing KARs during tetanization is sufficient to induce MF-LTP in vivo, such that all three receptors need to be antagonized for the process to be blocked. This suggests that all three receptor subtypes are activated synaptically during high-frequency transmission and play interchangeable roles in the induction of MF-LTP in vivo.

## MATERIALS AND METHODS

This study was performed in accordance with The Animals (Scientific Procedures) Act (1986) and with the approval of the University of Bristol Ethics Committee.

### Electrophysiology

Adult male Wistar rats (300–400 g; Charles River Laboratories, United Kingdom) were anesthetized with a sodium pentobarbitone solution (60 mg/kg, i.p.; Sigma-Aldrich, United Kingdom). The jugular vein was then cannulated with PE10 tubing and anesthesia was maintained by continual intravenous perfusion of sodium pentobarbitone (≈20 mg/kg/h). Surgical anesthesia was confirmed by the absence of the pedal withdrawal and corneal reflexes. Urethane was used to anesthetize animals in a subset of experiments (1.4 g/kg, i.p.; 25% urethane). The anesthetized rat was placed in a stereotaxic frame and its temperature was maintained at 37 °C with a homeothermic blanket.

**Figure 1:**
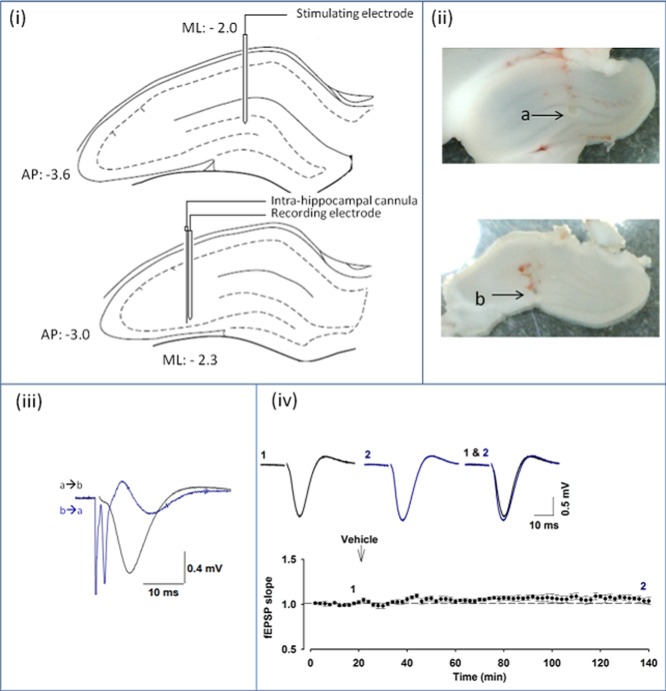
Mossy fiber LTP in vivo. (i) Schematic illustration of stimulating (upper) and recording electrode (lower) positions in the coronal plane of the hippocampus with corresponding stereotaxic co-ordinates (adapted from Paxinos and Watson's Atlas, The Rat Brain in Stereotaxic Coordinates, 6th ed., 2006, Elsevier.). (ii) Examples of electrically induced lesions illustrating stimulating (a) and recording (b) electrode positions in the hippocampus from a MF-LTP experiment, (iii) fEPSP elicited by stimulating from dentate gyrus (a) to CA3 (b); Spike evoked by antidromic stimulation of the granule cells in CA3 (b) and recorded in dentate gyrus (a), (iv) fEPSP recordings remain stable for >140 min (*n* = 5). Vehicle injection (i.h.) has no effect on fEPSP slope. Insets are representative fEPSPs at the indicated time points from individual experiments. [Color figure can be viewed in the online issue, which is available at wileyonlinelibrary.com.]

Stimulating and recording electrodes were then positioned in the MF pathway. A concentric bipolar tungsten electrode was positioned in the granule cell layer of the dentate gyrus according to the co-ordinates from bregma (anterior–posterior [AP], −3.6; mediolateral (ML), −2.0). A further concentric bipolar tungsten electrode was positioned in area CA3 of the hippocampus (AP, −3.0; ML, −2.3) (Fig. 1(i)). While using it to stimulate antidromically, the electrode in area CA3 was lowered until characteristic spikes from the granule cells, with a peak onset of 2–3 ms, were observed from the electrode in the dentate gyrus (Derrick and Martinez, 1994). The depth of the electrode in the dentate gyrus was then adjusted until characteristic MF field excitatory postsynaptic potentials (fEPSPs) were achieved following orthodromic stimulation (Fig. 1(iii)). These fEPSPs had an onset latency of ≈2–3 ms and a peak of ≈7–10 ms (Derrick and Martinez, 1994). (2*S*,2′*R*,3′*R*)-2-(2′,3′-dicarboxycyclopropyl)glycine) (DCG-IV) was applied at the end of all MF experiments to confirm the absence of associational/commissural pathway contamination (Kamiya et al., 1996; Yeckel et al., 1999; Hagena and Manahan-Vaughan, 2010; Sherwood et al., 2012). The experiments where DCG-IV did not depress the fEPSP slope by at least 70% were excluded from any analysis, for the concern of contamination from another form of LTP.

The stimulating electrode positioned in the dentate gyrus was connected to a constant current isolated stimulator (Quest scientific, Digitimer), which in turn was attached to a personal computer via a digital analogue converter (National Instruments, BNC2090). Stimulation intensity was set manually on an isolated stimulator, with a single, biphasic stimulus with the duration of 0.2 ms and intensity initially at 10 μA. The responses were obtained through the recording electrode, amplified with a differential amplifier (Warner Instruments, PP-304) and were filtered at 0.1 Hz–1 kHz. In brief, 50 Hz noise was minimized with a Hum Bug (Quest Scientific, Digitimer). The recordings were processed and stored via an interface digital/analogue converter board (M-series, v1.01) to a personal computer (Windows XP) running Win LTP (Anderson and Collingridge, 2007).

The stimulation intensity was set to evoke fEPSPs of ∼50% of the maximal response and a baseline frequency of 0.033 Hz was used throughout. The initial fEPSP slope (12.5–87.5% of the rising phase) was used as a measure of response magnitude. After obtaining a stable baseline, of at least 15 min, antagonists were applied over a 10 min period by intrahippocampal injection. To achieve this, a guide cannula was attached to the recording electrode. A 33 gauge cannula was inserted through the guide, with the end positioned 1 mm adjacent to the recording electrode tip. The cannula was connected to PE10 tubing, drug solutions were drawn into the tubing, as measured with a 5 μL microsyringe (Hamilton). In most experiments, 20 min after the i.h. injection, ketamine was injected (10 mg/kg, i.v.). Two trains of tetanization were then applied (100 Hz, 1 s, 30 s intertrain interval) 10 min later.

At the completion of every experiment, high-intensity current was passed through both the stimulating and the recording electrodes. The resulting lesions allowed the determination of the electrode positions. The animal was then sacrificed by cervical dislocation and the brain was removed. The cerebellum and frontal cortex were removed and the brain was mounted for coronal slicing. The mounted tissue was bathed in physiological saline (0.9%). Slices (thickness, 300 μm) were cut, removed, and stored in physiological saline to avoid swelling or dehydration. The photographs of the lesions were taken, from unstained tissue, with a universal serial bus Microcapture camera and software (Fig. 1(ii)). The positioning of electrodes was used as another criterion to confirm that fEPSPs were originating from the MFs. The experiments in which the stimulating electrode was not in dentate gyrus or the recording electrode was not approximately in stratum lucidum of CA3 were discarded.

### Drugs

Stock solutions for all drugs applied by i.h. injection were diluted in physiological saline (0.9%). ACET, d-(−)-2-amino-5-phosphonopentanoic acid (D-AP5), DCG-IV, LY367385, MCPG, and MPEP were all sourced from Tocris, Untied Kingdom; sodium pentobarbitone and urethane were obtained from Sigma-Aldrich, United Kingdom; UBP161 was synthesized in-house as reported previously (Irvine et al., 2012). The compounds, ACET (0.1 nmol), d-AP5 (1 nmol), UBP161 (1 nmol), LY367385 (0.3 and 1 nmol), MPEP (0.3 and 1 nmol), MCPG (1, 5 and 50 nmol), and interleaved vehicle controls, were injected in a 2 μL volume i.h. over a 10 min period. DCG-IV (0.01 nmol) was injected in a 1 μL volume i.h. over a 10 min period, 90 min after tetanization. The concentrations of mGlu agonist or antagonists were similar to those used by others (Manahan-Vaughan and Reymann, 1997; Manahan-Vaughan et al., 1998; Naie and Manahan-Vaughan, [Bibr b34],[Bibr b35]).

### Data Analysis

All data were presented as the fEPSP slope normalized to baseline, with each point representing the average of four successive fEPSPs recorded at 30 s intervals. Pooled data were presented as the mean of the normalized fEPSPs of individual experiments ± the standard error of the mean.

One-way ANOVAs, with Dunnet's post hoc tests, were used to compare the magnitude of MF-LTP (at 90 min after tetanization) in rats treated with each antagonist versus the interleaved vehicle-treated control group. For the experiments investigating the NMDAR dependence of CA1 LTP, the magnitude of LTP 60 min after tetanization was compared, in the presence or absence of d-AP5, with a *t*-test.

## RESULTS

### Properties of MF-LTP In Vivo

**Figure 2:**
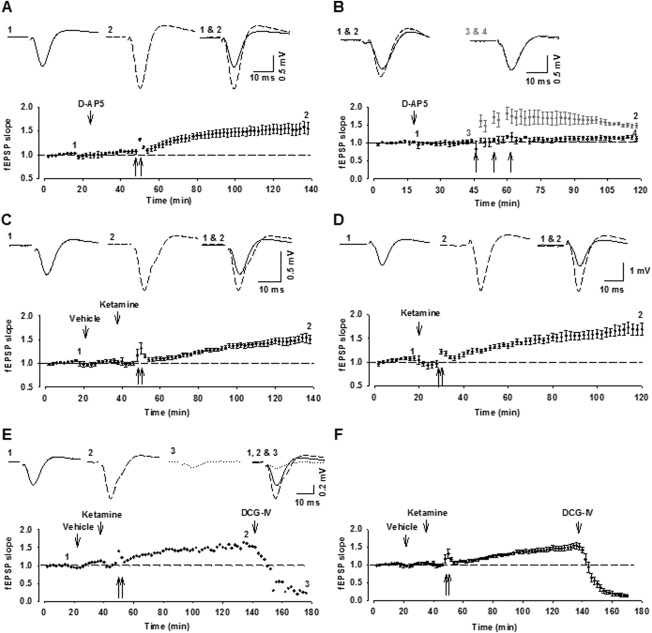
The properties of MF-LTP in vivo. (A) MF-LTP can be induced in the presence of the NMDAR antagonist, D-AP5 (1 nmol, i.h.; *n* = 4). (B) CA1 LTP can be induced by three trains of tetanization (3 × 100 stimuli at 100 Hz, 240 s intertrain interval; *n* = 4). The application of D-AP5 (1 nmol, i.h.) 30 min before tetani blocks CA1 LTP (*n* = 4, darker symbol). (C) MF-LTP recorded in the presence of ketamine (10 mg/kg, i.v., 10 min before tetani). (D) Slow-onset MF-LTP is also induced with urethane anesthesia (*n* = 4). (E, F) A single example (E) and pooled data (F) (*n* = 11) showing the effects of DCG-IV (0.01 nmol, i.h.), tested 90 min after the induction of MF-LTP. Unless otherwise stated, in this and subsequent figures, gray symbols represent vehicle controls, and black symbols represent the drug treatment indicated. In this figure and Figures 3 and 4, traces are the average of 4 consecutive fEPSPs responses obtained during baseline (1) and 90 min following (2) the delivery of two tetani (arrows).

In the presence of the NMDAR antagonist, D-AP5 (i.h.), high-frequency stimulation (HFS) (2 × 100 Hz for 1 s, 30 s intertrain interval) within the dentate gyrus, resulted in a slow-onset LTP recorded in area CA3 (152 ± 12% of baseline, 90 min after tetanization; *n* = 4; Fig. 2A). The effectiveness of the D-AP5 treatment was evaluated in area CA1. In this region, HFS (3 × 100 Hz, 1 s, 240 s intertrain interval) induced a rapid-onset LTP (148 ± 6%; *n* = 4, gray symbols) that was significantly inhibited by the NMDAR antagonist (112 ± 7%; *n* = 4, black symbols; *t*-test, *P* < 0.05; Fig. 2B).

A slow-onset LTP was also observed in area CA3 in rats in which the NMDAR antagonist ketamine (Anis et al., 1983) was injected (10 mg/kg, i.v.) 10 min before delivering HFS (155 ± 5%, *n* = 11; *P* > 0.05; Fig. 2C). These data demonstrate that HFS can induce an NMDAR-independent LTP in area CA3 after stimulation within the dentate gyrus. Although this LTP differed from MF-LTP as observed in slice preparations by the absence of short-term potentiation (STP) (Contractor et al., 2001; Lauri et al., 2001), it closely resembled that observed previously in anesthetized rats (Derrick and Martinez, 1994; Thompson et al., 2005; Gomez-Palacio and Escobar, 2012). In all subsequent experiments, ketamine (10 mg/kg, i.v.) was applied prior to the delivery of HFS.

We wondered if the absence of STP might be owing to the use of a barbiturate anesthetic. However, a similar slow onset LTP was observed when anesthesia was induced with urethane (182 ± 16%, *n* = 5; Fig. 2D).

### MF fEPSPs Are Sensitive to Group II mGluR Agonism

The application of a Group II mGluR agonist, such as DCG-IV, is often used to define mossy fiber projections (Kamiya et al., 1996; Mellor and Nicoll, 2001; Gomez-Palacio and Escobar, 2012; Hagena and Manahan-Vaughan, 2010). Here, MF fEPSPs were found to be sensitive to DCG-IV (0.01 nmol, i.h.) (Fig. 2E). DCG-IV reduced the fEPSP slope relative to preinjection levels (reduction, 91 ± 3%; *n* = 11; *P* < 0.05; Fig. 2F).

### Inhibition of Either KARs or mGluRs Alone Does Not Affect MF-LTP

**Figure 3:**
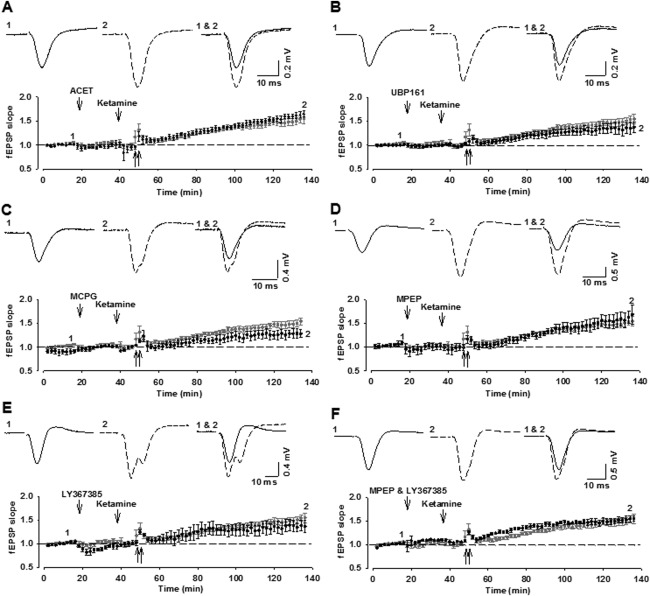
KAR or mGluR antagonists applied alone do not affect MF-LTP. (A, B) The GluK1 antagonists ACET (A: 0.1 nmol, i.h.; *n* = 5) or UBP161 (B, 1 nmol, i.h.; *n* = 5) fail to block MF-LTP. (C–F) The broad spectrum mGluR antagonist MCPG (C, 5 nmol, i.h.; *n* = 5), the mGlu5 receptor antagonist, MPEP (D, 1 nmol, i.h.; *n* = 4), the mGlu1 receptor antagonist LY367385 (E, 1 nmol, i.h.; *n* = 4) or the combination of LY367385 and MPEP (F, 1 and 1 nmol, i.h.; *n* = 4) does not block MF-LTP.

We started by testing two KAR antagonists, ACET and UBP161, which have been shown to be potent at GluK1-containing KARs (Dargan et al., 2009; Irvine et al., 2012). We applied the concentrations that we considered would substantially block GluK1-containing KARs in vivo, based on their established potencies as GluK1 antagonists and the dilution factor estimated from the DCG-IV experiments. However, we were still able to induce MF-LTP following i.h. injections of either of these compounds: ACET (0.1 nmol; 159 ± 7%; *n* = 5; Fig. 3A) or UBP161 (1 nmol; 137 ± 12%; *n* = 5; Fig. 3B).

Next, we tested three mGluR antagonists, MCPG (Eaton et al., 1993), MPEP (Gasparini et al., 1999), and LY367385 (Clarke et al., 1997), using the concentrations that we estimated should effectively engage their targets based on their known potencies. For each antagonist we used two different doses. Invariably, we were also able to induce MF-LTP: MCPG (5 nmol: 126 ± 7%; *n* = 5; Fig. 3C; 50 nmol: 137 ± 11%; *n* = 5), MPEP (0.3 nmol: 135 ± 6%; *n* = 4; 1 nmol: 168 ± 21%; *n* = 4; Fig. 3D), LY367385 (0.3 nmol: 159 ± 22%; *n* = 3; 1 nmol: 137 ± 11%; *n* = 4; Fig. 3E). We also observed LTP when we applied a combination of MPEP plus LY367385 (1 nmol of each: 155 ± 6%; *n* = 4; Fig. 3F). In all cases, there was a significant LTP that was not significantly different to that induced in the vehicle-treated control groups (*P* > 0.05).

### Effects of the Combined Inhibition of mGluRs and KARs on MF-LTP

**Figure 4:**
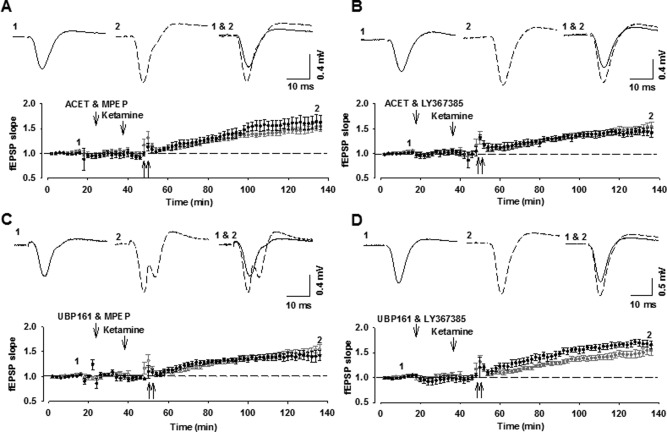
Combined application of KAR antagonists with either mGlu1 or mGlu5 receptor antagonists does not affect MF-LTP. (A–D) Intrahippocampal injection of ACET and MPEP (A: 0.1 and 1 nmol, i.h.; *n* = 4), UBP161 and MPEP (B: 1 and 1 nmol, i.h.; *n* = 4), ACET and LY367385 (C: 0.1 and 1 nmol, i.h.; *n* = 4) or UBP161 and LY367385 (D: 1 and 1 nmol, i.h.; *n* = 4) does not affect MF-LTP.

Next, we tested the effects of inhibiting either mGlu1 or mGlu5 together with the inhibition of KARs. With all combinations, HFS again elicited a significant MF-LTP that was not significantly different to that induced in vehicle-injected controls (0.1 nmol ACET plus 1 nmol MPEP; 162 ± 15%; *n* = 4; 1 nmol UBP161 + 1 nmol MPEP: 142 ± 10%; *n* = 4; 0.1 nmol ACET + 1 nmol LY367385: 145 ± 14%; *n* = 4; 1 nmol UBP161 + 1 nmol LY367385: 166 ± 8%; *n* = 4; *P* > 0.05; Fig. 4).

**Figure 5:**
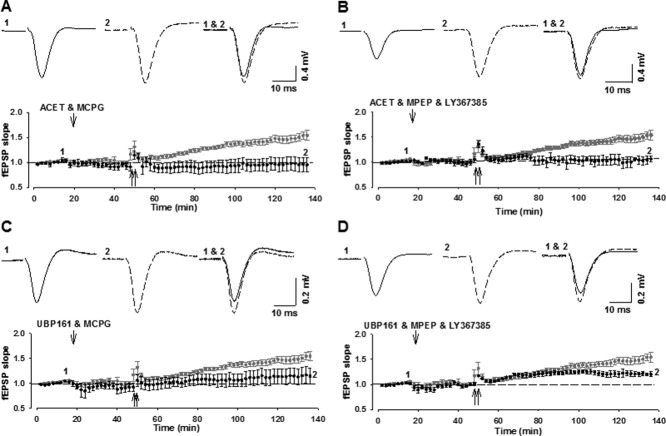
Combined application of KAR antagonists with mGlu1 and mGlu5 receptor antagonists inhibits MF-LTP. (A–D) Intrahippocampal injection of ACET and MCPG (A: 0.1 and 5 nmol, i.h.; *n* = 4), ACET and MPEP and LY367385 (B: 0.1, 1, and 1 nmol, i.h.; *n* = 4) UBP161 and MCPG (C: 1 and 5 nmol, i.h.; *n* = 5) or UBP161 and MPEP and LY367385 (D: 1, 1 and 1 nmol, i.h.; *n* = 5) blocks the induction of MF-LTP.

**Figure 6:**
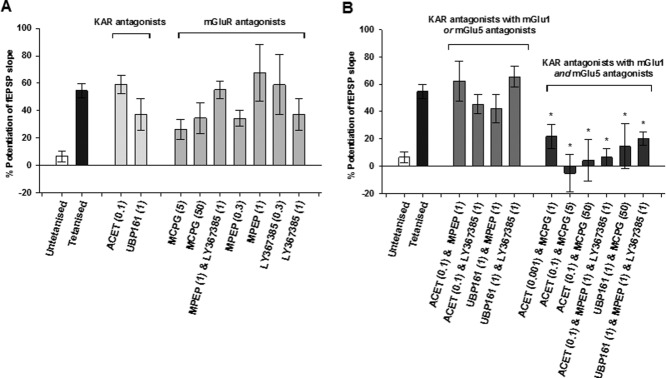
Summary of the effects of KAR and group 1 mGluR antagonists on MF-LTP magnitude. (A) No significant effects of KAR or mGluR antagonists on MF-LTP when applied alone. (B) No significant effects of KAR antagonists when applied with an mGlu1 or with an mGlu5 receptor antagonist. But the combination of KAR and mGlu1 and mGlu5 receptor antagonists did significantly reduce MF-LTP (*P < 0.05). The control from all interleaved experiments is shown in both (A) and (B) for the convenience of the reader. The numbers in parenthesis are the drug doses in nmol.

Finally, we tested the effects of inhibiting mGlu1 and mGlu5 together with KARs. First, we tested the effects of ACET along with three doses of MCPG and the combination of MPEP and LY367385. In all cases, LTP was significantly smaller compared to vehicle or eliminated (0.001 nmol ACET + 1 nmol MCPG: 122 ± 9; *n* = 5; 0.1 nmol ACET + 5 nmol MCPG: 95 ± 14%; *n* = 4; *P* < 0.05; 0.1 nmol ACET + 50 nmol MCPG: 104 ± 15; *n* = 4; 0.1 nmol ACET + 1 nmol MPEP + 1 nmol LY367385: 107 ± 6%; *n* = 4; *P* < 0.05; Figs. 5A,B). Similar effects were observed with UBP161 (1 nmol UBP161 + 50 nmol MCPG: 137 ± 16%; *n* = 5; *P* < 0.05; 1 nmol UBP161 + 1 nmol MPEP + 1 nmol LY367385: 120 ± 5%; *n* = 5; *P* < 0.05; Figs. 5C,D). These data suggest that HFS of MFs results in the activation of all of the three receptors studied (mGlu1, mGlu5, and KAR); such that both Group I mGluRs plus GluK1-KARs need to be antagonized to prevent MF-LTP in vivo. These data are summarised in Figure 6.

## DISCUSSION

In this study, we have examined the sensitivity of MF-LTP in vivo to a variety of mGluR and KAR antagonists. Our principal finding is that the activation of either one of the Group I mGluRs (mGlu1 or mGlu5) or a KAR is sufficient for the induction of MF-LTP in anesthetized rats. Thus, it was necessary to block both mGlu1 and mGlu5 as well as KARs to prevent the induction of MF-LTP. These observations show that glutamate receptors are required for the induction of MF-LTP but that KAR and mGluRs can play interchangeable roles.

### MF-LTP In Vivo

The LTP that we recorded in this study did not resemble MF-LTP as observed in hippocampal slices in that we observed a slowly developing potentiation. In these respects, the LTP that we observed closely resembled MF-LTP as initially described in anesthetized rats by Derrick et al. (1991). Despite the differences from the extensive number of studies performed in slices, we are confident that we were studying MF-LTP because (i) we only observed this LTP when the electrodes were correctly positioned to stimulate and record from the MF pathway, (ii) this LTP was resistant to antagonism of NMDA receptors, and (iii) the recorded fEPSPs were reduced by DCG-IV. We do not know the reason for the absence of STP, but it is unlikely to be attributed to the use of any one specific anesthetic as we observed a similar profile when using either pentobarbitone or urethane. A similar profile has also been observed with isoflurane (Ballesteros et al., 2012). Other possibilities include the differences in temperature between in vitro (typically between ∼22 and 31 °C: Pinheiro et al., 2007; Nistico et al., 2011) and in vivo (37 °C) preparations, and the differences in divalent cation concentrations.

### Glutamate Receptors and the Induction of MF-LTP In Vivo

We tested three different mGluR antagonists. MCPG is the prototypic mGluR antagonist (Eaton et al., 1993) that inhibits some (mGlu1, mGlu2, mGlu3, mGlu5, and mGlu8) but not all mGluR subtypes (Schoepp et al., 1999). MPEP is a potent and selective mGlu5 receptor antagonist (Gasparini et al., 1999), with weak activity at NMDA receptors in high concentrations (O'Leary et al., 2000). LY367385 is a fairly selective mGlu1 receptor antagonist (Clarke et al., 1997). Our finding that neither LY367385 nor MPEP alone or in combination significantly affected LTP suggests that the inhibition of Group I mGluRs alone is not sufficient to block LTP. Consistent with this conclusion, MCPG also failed to prevent the induction of MF-LTP. These observations contrast with the report that a different Group I mGluR antagonist, AIDA, can prevent the induction of MF-LTP in vivo (Thompson et al., 2005). It is possible, however, that the differences in the experimental conditions *in vivo* can affect the ability of Group I mGluR antagonists to affect MF-LTP. In this study, it is unlikely that the antagonists failed to reach the concentrations effective for antagonizing Group I mGluRs as they were highly effective when applied in combination with KAR antagonists. We conclude, therefore, that MF-LTP can be induced in vivo despite substantial inhibition of Group I mGluRs.

We tested two structurally different KAR antagonists. ACET is a highly potent antagonist at GluK1-containing KARs (Dargan et al., 2009) and has weaker activity at some GluK3-containing KARs (Perrais et al., 2009). UBP161 is a more recently described KAR antagonist that is not related structurally to ACET (Irvine et al., 2012). It is less potent, but more selective, than ACET as a GluK1 antagonist, displaying over a 100-fold selectivity at GluK1 relative to GluK2 and GluK3 (Irvine et al., 2012). It is also an NMDA receptor antagonist (Irvine et al., 2012). Our finding that neither ACET nor UBP161 affected LTP suggests that the inhibition of GluK1-containing KARs alone is not sufficient to prevent LTP in vivo. Again, their effectiveness in combination with mGluR antagonists argues against the possibility that we did not achieve a sufficiently high concentration to antagonize KARs.

The finding that the combinations of mGluR and KAR antagonists were effective at blocking MF-LTP argues for an involvement of both ionotropic and metabotropic receptors in this process. As we observed similar effects using either MCPG or a combination of MPEP and LY367385 and similar effects using ACET or UBP161 it is unlikely that the sites of action are some undefined target. Rather, we would argue that these results strongly suggest the need to antagonize both Group I mGluRs and KARs to prevent the induction of LTP. Interestingly, it was necessary to block both mGlu1 and mGlu5, suggesting that these play interchangeable roles. Surprisingly, the observation that it was additionally necessary to block KARs suggests that mGluRs and KARs play interchangeable roles too. This is an unusual scenario where metabotropic and ionotropic glutamate receptors can substitute for one another in a physiological function.

### Comparison with Studies in Hippocampal Slices

How do our findings in vivo compare with those in hippocampal slices? In making this comparison, it is important to note that there are striking differences in the physiology and pharmacology of MF responses and LTP profiles between parasagittal and transverse slices (Sherwood et al., 2012).

With respect to synaptic waveforms, the responses that we have recorded are similar to those obtained from parasagittal slices but quite distinct from those observed in transverse slices, which tend to be much smaller, faster, and irregular in appearance. In terms of mGluRs, our findings that neither MPEP nor LY367385 blocked LTP when applied alone are consistent with our previous studies in parasagittal brain slices using the same antagonists (Nistico et al., 2011). However, in contrast to this study, we observed complete block of MF-LTP when we used either MCPG (Bashir et al., 1993; Nistico et al, 2011) or a combination of MPEP and LY367385 (Nistico et al., 2011) in parasagittal slices. The lack of effect of MCPG, that we have observed in this study, resembles the situation in experiments that have used transverse hippocampal slices (Manzoni et al., 1994; Hsia et al., 1995). The effects of DCG-IV are similar to those reported by us (Sherwood et al., 2012) and others (Kamiya et al., 1996) using transverse slices but differ from our observations in parasagittal slices where responses were insensitive to this group II mGluR agonist.

With respect to KARs, the finding that ACET when applied alone had no effect on LTP is consistent with our observations in transverse slices but contrasts with our findings in parasagittal slices, where ACET fully blocked LTP (Dargan et al., 2009; Sherwood et al., 2012). Indeed, when using parasagittal brain slices, we have observed the block of LTP by six structurally distinct KAR antagonists over a 20,000-fold concentration range (Jane et al., 2009).

In summary, the nature of the MF-LTP observed in this study neither matches that seen by us or others in either transverse or parasagittal slices, but has some features in common with both. It is most similar to our previous work in parasagittal slices, where we found that either mGlu1 and mGlu5 antagonism or GluK1 antagonism was sufficient to block the induction of MF-LTP (Nistico et al., 2011). The principal difference being that here we found that mGlu1 and mGlu5 plus GluK1 antagonism is required.

### The Mechanism of Induction of MF-LTP In Vivo

Based on our present findings, we would suggest that both mGluRs and KARs are involved in MF-LTP in vivo. Our results are consistent with a scenario where any of the three receptors studied, mGlu1, mGlu5, or GluK1-KARs, is sufficient to induce MF-LTP and hence the need to antagonize all three. The major difference between the present observations and our previous work in vitro is the need to block KARs and Group I mGluRs as opposed to either type alone. One potential explanation for this difference could be at the level of Ca^2+^ stores. We have shown that in parasagittal slices, the release of Ca^2+^ from ryanodine-sensitive stores is required for MF-LTP (Lauri et al., 2003). We speculated that Group I mGluRs, which couple to phospholipase C, generate IP_3_ and KARs lead to Ca^2^ entry, which then act in concert to release Ca^2^ from stores. The present results could be explained by a similar mechanism, but where *either* IP_3_, generated by the activation of mGlu1 and/or mGlu5 receptors, *or* Ca^2+^ entry, resulting from the activation of KARs, is sufficient to release Ca^2+^ from stores. This might occur if stores are in a more sensitized state in vivo, possibly as a result of a more complete filling of the stores under resting conditions. Further work will be required to test this hypothesis directly.
